# The Immunomodulatory Capacity of an Epstein-Barr Virus Abortive Lytic Cycle: Potential Contribution to Viral Tumorigenesis

**DOI:** 10.3390/cancers10040098

**Published:** 2018-03-30

**Authors:** Abigail Morales-Sánchez, Ezequiel M. Fuentes-Panana

**Affiliations:** Research Unit in Virology and Cancer, Children’s Hospital of Mexico Federico Gómez, Mexico City 06720, Mexico; abimor2002@yahoo.com.mx

**Keywords:** EBV, KSHV, HCMV, abortive lytic cycle, immunomodulation, tumorigenesis, autocrine/paracrine signaling, oncomodulation

## Abstract

Epstein-Barr virus (EBV) is characterized by a bipartite life cycle in which latent and lytic stages are alternated. Latency is compatible with long-lasting persistency within the infected host, while lytic expression, preferentially found in oropharyngeal epithelial tissue, is thought to favor host-to-host viral dissemination. The clinical importance of EBV relates to its association with cancer, which we think is mainly a consequence of the latency/persistency mechanisms. However, studies in murine models of tumorigenesis/lymphomagenesis indicate that the lytic cycle also contributes to cancer formation. Indeed, EBV lytic expression is often observed in established cell lines and tumor biopsies. Within the lytic cycle EBV expresses a handful of immunomodulatory (*BCRF1*, *BARF1*, *BNLF2A*, *BGLF5* & *BILF1*) and anti-apoptotic (*BHRF1* & *BALF1*) proteins. In this review, we discuss the evidence supporting an abortive lytic cycle in which these lytic genes are expressed, and how the immunomodulatory mechanisms of EBV and related herpesviruses Kaposi Sarcoma herpesvirus (KSHV) and human cytomegalovirus (HCMV) result in paracrine signals that feed tumor cells. An abortive lytic cycle would reconcile the need of lytic expression for viral tumorigenesis without relaying in a complete cycle that would induce cell lysis to release the newly formed infective viral particles.

## 1. Introduction

Epstein-Barr virus (EBV) belongs to the Herpesviridae, a family of large enveloped viruses with a double stranded DNA genome, which exists in the nucleus of infected cells as pieces of extrachromosomal episomal DNA. Another distinguishing feature of EBV and the Herpesviridae is their bipartite life cycle in which latent and lytic stages are alternated allowing the viruses to persist for the whole life of the infected host. The medical importance of EBV lies in its association with several human malignancies, mainly B cell lymphomas in both immunocompetent and immunocompromised individuals, carcinomas of the stomach and nasopharynx, and more rarely with (natural killer) NK and T cell lymphomas and leiomyosarcomas. Infection is also associated with non-cancerous diseases such as infectious mononucleosis, chronic active EBV, and hemophagocytic lymphohistiocytosis, which sometimes also increase the risk of developing lymphoma [1,2].

A current model of EBV infection considers that the virus is transmitted orally, most likely in saliva droplets, infecting first the oropharyngeal mucosa. Evidence exists for the capacity of the virus to infect both B cells and epithelial cells, but while the former are easily infected and immortalized in vitro, infection of the latter is poorly efficient and needs particular experimental conditions, such as addition of IgA immunoglobulin, epithelial cell polarization with viral exposure through the basolateral face and B cell-epithelial cell direct interactions [3,4,5,6,7,8,9]. Also, in vitro infected epithelial cells tend to lose the viral episomes upon serial passages [6,10,11]. These data support that B cells are the main targets of EBV infection and the main reservoir of persistent latent infection, while infection of epithelial cells is more important for viral transmission to new hosts, for which the viral lytic cycle is essential. Nevertheless, the capacity of EBV to oscillate between B cells and epithelial cells, and between latent and lytic cycles facilitates viral persistence ad infinitum within the host and makes EBV one of the most prevalent viruses in the world population.

We still know little about the balanced interactions between EBV and host during the course of a long-lasting asymptomatic infection and the number of viral strategies required for successful persistence. Analysis of viral gene expression in associated cancers is compatible with a model in which EBV is also able to oscillate between several types of latent gene expression programs (latency 0, I, II and III), in which different sets of viral genes are expressed, including nuclear proteins (EBNA-1, EBNA-2, EBNA-3a, -3b and -3c and EBNA-LP (leader protein)), latent membrane proteins (LMP1, LMP2A and 2B), non-coding RNAs (EBER1 and EBER2) and about 44 micro RNAs (miRNAs). According to the germinal center model of infection, EBV has evolved with these patterns of latent gene expression to exploit the B cell maturation pathway leading infected B cells into the long-lived memory pool [12]. B cells express all latent proteins upon infection (latency III) and progressively downregulate their expression as the infected cell differentiates into a germinal center centrocyte/centroblast cell to finally reach the memory stage. On the contrary, the lytic cycle is characterized by extensive viral gene expression, harboring about 80 additional proteins. The viral lytic cycle is divided into three temporal and functional stages: immediate early (IE), early (E) and late (L). IE gene products are transcription factors in charge of turning on the cascade of expression of lytic genes; expression of E genes depends on de novo translation of IE transcription factors but it is not affected by inhibitors of DNA synthesis, while expression of L genes is significantly more efficient after extensive replication of the viral genome. Hence, E genes mainly encode enzymes with DNA replication function and L genes are mostly viral structural proteins.

The switch from a latent to lytic infection is termed viral reactivation, and BZLF1 (also known as Zta, ZEBRA or Z protein) and BRLF1 (also known as Rta) are the IE transcription factors that master regulate EBV reactivation/lytic expression. Although there are many known stimuli used in vitro to reactivate EBV, the most physiological signal encountered within the host most likely is a cognate antigen. Considering that EBV resides in quiescent long-lived memory B cells, immunogenic stimuli that switch those cells to become finite antibody-producing terminally differentiated plasma cells would upset viral latency activating mechanisms leading to viral exit of the antigen-activated B cell. Multiple signaling pathways are turned on upon activation of the B cell antigen receptor (BCR), and several transcription factors downstream of the BCR, including Jun/Fos heterodimer (Activator Protein 1 (AP1)), initiate transcription from the *BZLF1* promoter. Zta exhibits functional and structural homology with AP1 and regulates its own and Rta expression through AP1-like binding sites located in the EBV lytic E promoters. Furthermore, activation of the BCR-induced phospholipase C gamma (PLCγ) and mitogen-activated protein kinase (MAPK) pathways is widely mimicked in the laboratory to reactivate EBV, through the use of the 12-*O*-tetradecanoylphorbol-13-acetate (TPA/PMA) phorbol ester. PLCγ activates protein kinase C (PKC), which in turn phosphorylates Zta at Ser-186, an event essential for Zta transcriptional activation [13]. Other common reagents used to reactivate EBV are BCR crosslinking antibodies, histone deacetylases (HDACs), DNA methyltransferase inhibitors and transforming growth factor-β (TGF-β). Also, several inducers of cellular stress, such as inducers of DNA damage and reticulum endoplasmic stress, hypoxia and inflammation through cyclooxygenase-2 (COX-2)/prostaglandin E2 (PGE2) [13]. Although epithelial cells do not express an antigen receptor, inducers of cell differentiation have also been shown to reactivate EBV infected epithelial cells [14]. Indeed, some of the transcription factors that participate in the memory to plasma B cell differentiation have also been shown to participate in epithelial cell differentiation [15].

## 2. An Abortive Lytic Cycle in Pre-Latent Cells and Established Tumors

Although the great clinical importance of EBV relates to its association with cancer, cancer is not the goal of viral infection as implies the end of both virus and host. Instead, cancer is an unwanted side effect of the viral mechanisms of persistence. Traditionally, we have conceived the latent and lytic viral cycles as two mutually exclusive mechanisms that together contribute to EBV persistency at the individual and population levels. The restrictive mode of latent viral gene expression is more compatible with the individual long-lasting persistency because it allows EBV to hide from antagonistic host immune responses. Thus, the most unrestrictive latent expression (latency III) is observed mainly in immunocompromised individuals and upon in vitro B cell infection, while in asymptomatic immunocompetent hosts EBV is found in a latency 0 program in which there is no expression of viral proteins [16]. On the contrary, lytic expression is preferentially found in oropharyngeal epithelial tissue in which it is thought to promote the release of infective viral particles to saliva. However, there is evidence that sometimes both latent and lytic gene expression patterns coincide within the same cell. The study by Wen W. et al. was the first to report expression of *BZLF1* in freshly infected B cells [17]. *BZLF1* expression was observed as early as 0.5 h after infection and lasting for several days. In contrast, expression of *EBNA-2*, the master regulator of latency III and of B cell immortalization, was observed at six hours post infection. Zta protein was confirmed by Western blot analysis. Interestingly, expression of the late gene *BLLF1* (encodes for gp350/220) was not detected indicating an only partial activation of the lytic cycle.

Zta is a transcription factor that preferentially binds and transactivates methylated promoters [18]. Extensive EBV genome methylation occurs upon establishment of latency. Although it is believed that this epigenetic modification is essential to control lytic gene expression, it is not an impediment for Zta to initiate transcription of IE and E lytic genes upon viral reactivation (reviewed in [19]). Kalla, M. et al. proposed that the ability of Zta to induce a full lytic cycle might depend on the level of methylation of the viral genome [20]. This group observed that after infection, when the viral genome is not methylated, while Zta was unable to lead to the production of progeny virus, it was still capable to bind many viral promoters acting as a mitogenic signal to the newly infected naïve B cell. Hence, lytic cycle expression was not limited to Zta but also to other early genes but not to late structural proteins. Expression of selected lytic genes was observed at least for ten days after infection. A similar observation was also made in EBV infected epithelial cells, in which both *BZLF1* mRNA and protein were observed soon after infection that also lasted for several days [21]. Tsang, C.H. et al. using telomerase-immortalized nasopharyngeal epithelial cells infected with EBV were also able to observe a transient pick of expression of *BZLF1* in the absence of *BLLF1* expression [22]. The expression of *BZLF1* with absence of other lytic genes, particularly those encoding late structural proteins, and thus, without formation of infective viral particles, is termed the abortive lytic cycle. When it occurs just after infection is known as the transient pre-latent abortive lytic cycle (reviewed in [23]). The contribution of Zta and other early lytic genes for establishment of viral latency is not clear, however, it was observed that a *BZLF1* knockout virus was unable to amplify the viral genome in infected AGS cells, a gastric carcinoma cell line, rendering Zta as a viral factor essential for genome replication [21], in addition to the mitogenic signal previously mentioned.

Evidence of an abortive lytic cycle in primary isolates of EBV positive cancer tissues has also been documented. Martel-Renoir, D. et al. in 1995 using RT-PCR detected expression of *BZLF1* in 8/8 nasopharyngeal carcinoma (NPC) biopsies [24]. Those samples expressed IE gene *BRLF1* and early gene *BMLF1*, but only 2/8 expressed the late structural gene *BLLF1*. Ramayanti O and colleagues reached a similar conclusion; they analyzed EBV gene expression in NPC biopsies and nasopharyngeal brushings from patients with suspected NPC. They consistently found high expression of IE genes *BZLF1* and *BRLF1*, and early gene *BGLF4* (encodes the EBV protein kinase), but low levels of other early genes (*BXLF1*, encodes the EBV thymidine kinase) and late genes (*BFRF3*, encodes the viral capsid antigen VCA p18), supporting the co-existence of latency and an abortive lytic cycle [25]. A transcriptomic analysis (RNAseq data) of eight EBV positive gastric cancers also showed a mixed expression of latent and lytic genes [26]. Xue, S. et al. also looked for *BZLF1* in 12 samples of Burkitt lymphoma (BL) isolated from Malawian children [14]. Evidence of mRNA expression was found in eight samples (66.7%). Zta expression was observed by immunohistochemistry in 6/7 samples tested and co-expression of latent and lytic genes at the same cell level was confirmed. Of note, other lytic genes were also expressed in BL samples, such as *BHRF1* (Bcl-2 viral orthologue), *BCRF1* (a viral orthologue of human interleukin-10) and *LF3*. Unfortunately, no late structural genes were addressed in this study and therefore it cannot be distinguished between a complete and an abortive lytic cycle. Also, *BZLF1* constitutive expression has been observed in 0.1–4% of cells within lymphoblastoid cell lines (LCLs) [27].

In a different type of study, Al Tabaa, Y. et al. isolated peripheral blood B cells from 13 EBV seropositive healthy individuals, which were stimulated in culture with CD40 ligand and a cytokine cocktail. They observed that 28.6% of the culture showed evidence of viral reactivation by expression of *BZLF1*, but evidence of full lytic cycle based on expression of *BLLF1* was only observed in 7% of cells, supporting that only a minority of reactivated B cells complete the lytic cycle [28]. The same group found a similar basal rate of abortive versus complete lytic cycle in B cells isolated from acute infectious mononucleosis patients [29]. Evidence of *BZLF1* expression without expression of the late gene *BDRF1* (encodes for VCA-p40) was found in the following EBV-associated disorders: systemic hydroa vacciniforme (HV), hypersensitivity to mosquito bites (HMB), and in the B, T and NK cell lymphomas that are associated with those diseases [30]. Importantly, a previous study from the same group had associated *BZLF1* expression with poor prognosis in the cutaneous lesions of HV and HBM [30].

## 3. Evidence That the Abortive Lytic Cycle Contributes with the EBV-Induced Tumorigenesis

There is convincing evidence that EBV lytic gene expression is required for optimal viral-induced tumorigenesis beyond a mere role in cell-to-cell viral dissemination. Ma SD and collaborators modeled a humanized mouse utilizing a nonobese diabetic/severe combined immunodeficient (NOD/LtSz-scid/IL2Rγnull, NSG) mouse, which was reconstituted with CD34+ human hematopoietic progenitor cells isolated from fetal liver, and also simultaneously xenotransplanted with fetal liver and thymus tissues [hNSG(thy)]. hNSG(thy) mice were infected with EBV developing CD20 positive diffuse large B cell lymphomas (DLBCL) [31]. To understand the contribution of the lytic cycle for lymphomagenesis, hNSG(thy) mice were infected with a *BZLF1* knockout EBV. Although no significant differences were apparent in the number of infected cells and in their tissue distribution, lymphomas were found in 6/11 (54.4%) of mice infected with wild type virus, and only in 2/14 (14.3%) of mice infected with the mutant virus. These data support that the lytic cycle directly helped tumorigenic mechanisms rather than the lateral spread of viruses that increases the number of latently infected cells. In agreement, lymphomas developing in mice infected with wild type viruses sometimes expressed Zta and BMRF1 lytic proteins but never gp350/220 structural protein. Hence, these data also support a participation of the abortive lytic cycle in lymphomagenesis. Hong, G. K. et al. made a similar observation; this group reported that when LCLs derived from wild type and virus knockout of *BZLF1* and *BRLF1* IE genes were transplanted into SCID mice, both mutant LCLs were deficient to induce lymphoproliferative disease (LPD) supporting a critical role for the lytic cycle in the EBV-induced lymphoproliferation [32]. Furthermore, wild type infected mice treated with acyclovir were not affected in their capacity to induce LPDs indicating that disease development was not dependent on viral DNA replication and hence on formation of infective viral particles. Taken together all the studies described above support that an abortive lytic cycle is transiently operative during the first days post infection, but also in chronically infected cells isolated from healthy individuals and cancer patients. Furthermore, this abortive lytic cycle seems to significantly contribute with the tumorigenic/lymphomagenic mechanisms of EBV.

## 4. Lytic Cycle Proteins with Anti-Apoptotic and Immunomodulatory Functions

Since lytic infection ends with the death of host cells, we have traditionally thought that the EBV-induced cell immortalization exclusively depends on latent infection. Therefore, the contribution of lytic cycle genes for cell transformation and viral tumorigenesis is to say the least, puzzling. However, mixed with the lytic transcription factors, viral DNA replication enzymes and structural proteins, there are a handful of genes encoding proteins with anti-apoptotic (BHRF1 & BALF1) and immunomodulatory (*BCRF1*, *BARF1*, *BILF1*, *BGLF5*, *BNLF2a*, *BLLF3* & *BPLF1*) functions that traditionally have been thought to enhance viral production by transiently protecting the lytically infected cells from endogenous death signals and exogenous immune responses (Table 1). The pathogenicity of these genes is not clear outside of their contribution to the generation of new viral particles and the spread of infection. Their dynamic expression as part of an abortive lytic cycle would reconcile them with a tumorigenic role, since these genes would still provide the immunomodulatory and anti-apoptotic assistance without the infected cell reaching the final lytic stages associated with cell death.

BHRF1 and BALF1 are viral orthologues of cellular Bcl-2 (B cell lymphoma 2) and as such it has been documented that BHRF1 inhibits the activity of pro-apoptotic proteins Bax and Bak, affecting cytochrome release and loss of the mytochondrion transmembrane potential [33]. BHRF1 also binds with high affinity to BH3-only proteins Bim (KD  =  18 nM), Puma (70 nM) and Bid (110 nM) [33]. In agreement, ectopic expression of BHRF1 in a mouse model of Burkitt lymphoma (Eµ-myc) conferred protection against the chemotherapeutic agents Ara-C, cyclophosphamide and etoposide [33]. BALF1 has been less studied but binding to Bax and Bak has also been described [34]. BHRF1 and BALF1 are known to be central for viral tumorigenesis. Experiments with mutant knockout viruses in one or both of these genes revealed redundant activities but an absolute requirement for at least one of them to transform primary B cells [35]. *BHRF1* and *BALF1* are highly expressed during the pre-latent abortive lytic cycle [35], but also their expression has been detected in BLs [14,36], DLBCLs [37] and in a subset of EBV associated gastric cancers (EBVaGC) [38,39].

Both latter studies also found expression of *BARF1* in most of the EBVaGC samples analyzed [38,39], and other RT-PCR based studies have also supported the expression of *BARF1* in EBV-induced epithelial malignancies [40,41]. Although up to today there is no definitive evidence of protein expression, anti-BARF1 antibodies have been detected in sera of NPC patients [42,43]. *BARF1* is one of the most abundant EBV transcripts in NPC cell lines, while in B cells and lymphomas it seems exclusively expressed after induction of the lytic cycle [44]. Most of the *BARF1* activities have been elucidated in transfection experiments with vectors encoding a recombinant protein, in which it has been documented that BARF1 is secreted as a soluble hexameric complex (sBARF1) in epithelial cells [45]. It is possible that detection of the protein is hampered by its secretory nature. Crystallographic analysis of sBARF1 showed a three-dimensional (3-D) structure similar to that of macrophage colony stimulating factor receptor (M-CSFR) immunomodulatory protein [46,47]. In agreement, BARF1 co-precipitates with the receptor’s ligand M-CSF and impaired the M-CSF-dependent macrophage phagocytic and oxidative activity [48,49]. Therefore, BARF1 functions as an antagonist of M-CSF interfering with monocyte differentiation and activation. Monocytes/macrophages are also important triggers of T cell activity through secretion of interferons, and monocytes isolated from peripheral blood failed to release IFN-α upon stimulation with TL3 ligands and sBARF1 [50]. Other cellular functions described for BARF1 are up-regulation of the anti-apoptotic protein Bcl-2, telomerase and cyclin D1 [51,52]. Indeed, ectopic expression of BARF1 also resulted in enhanced expression of cell proliferation and anti-apoptotic markers, and cells were protected against Taxol-induced apoptosis [53]. BARF1 immortalizes human epithelial cells [54,55]; therefore, together with LMP1, BARF1 is considered the other major EBV oncogene in NPC causation.

*BCRF1* encodes _ebv_IL-10, a viral orthologue of cellular interleukin-10, the quintessential immunosuppressive cytokine, so that it is also known as the cytokine synthesis inhibitory factor. As mentioned above, *BCRF1* is often co-expressed with *BZLF1* during abortive lytic expression, and in primary samples isolated from EBV positive carcinomas, and in B, T and NK cell lymphomas [38,39,56,57]. Recombinant expression of _ebv_IL-10 with a vaccinia viral vector resulted in reduced NK and cytotoxic T cell (CTL) responses speculated to help the establishment of latent persistence infection [58]. Indeed, a *BCRF1* antisense oligonucleotide affected the EBV capacity to immortalize B cells in vitro and to sustain post infection B cell growth [59], and cells infected with a *BCRF1* knockout virus were more efficiently eradicated, supporting the role of _ebv_IL-10 as part of the viral immune evasion mechanisms [60]. _ebv_IL-10 also downregulates the expression of TAP1 (peptide transporter associated with antigen processing 1) and the major histocompatibility complex-encoded proteasome subunit LMP2 affecting the transport of peptide antigens into de endoplasmic reticulum and their loading onto Human Leucocyte Antigen (HLA) class I molecules [61], and this presumably also contributes to viral evasion of CTL responses.

Like _ebv_IL-10, BILF1, BGLF5 and BNLF2a are three lytic cycle immunoevasins that also deregulate the host antigen-processing pathway to evade elimination of EBV infected cells. BILF1 expression has been detected in EBV LCLs and NPC cell lines [62]. BILF1 regulates surface levels of HLA class I molecules through direct binding leading to accelerated internalization and lysosomal degradation [63,64]. Indeed, BILF1 ectopic expression resulted in increased transforming capacity in a mouse xenograft tumor model [65]. *BILF1* is conserved among lymphocriptovirus isolated from non-human primates supporting an important pathological role [66]. BNLF2a prevented binding of TAP to both peptide and ATP, thereby inhibiting peptide loading onto HLA class I molecules and antigen presentation to CTLs [67,68,69]. Of note, while in B cells *BNLF2a* is expressed as a lytic cycle early phase antigen, in a subset of EBVaGCs is one of the most abundant viral transcripts, likely expressed together with the latency program [70,71]. BGLF5 is recognized as an alkaline exonuclease that shuts off the host’s cellular protein synthesis [72]. Although this function is more closely related to the capacity of EBV to globally eliminate cellular mRNAs to enhance translation of viral transcripts, shutting off protein synthesis significantly contributes to the EBV general mechanisms of immune evasion. Among the immune proteins downregulated upon BGLF5 expression are HLA class I and class II molecules and Toll-like Receptor 9 (TLR9), resulting in reduced levels of surface antigen presenting complexes and reduced T cell recognition of infected cells [72,73,74]. Lentiviral expression of *BGLF5* small hairpin RNAs (shRNAs) in EBV reactivated Akata B cells restored cellular levels of surface proteins TLR2, HLA class I and II molecules and CD1d [75]. Stronger T cell protection has been observed through knockdown experiments of the three EBV immunoevasins [76]. When 293T cells were transfected with bacterial artificial chromosomes carrying a *BGLF5* knockout EBV, it was found that viral production was 17- to 21-fold reduced [77]. Expression of *BGLF5* has been documented in NPC biopsies and antibodies to this immunoevasin have been detected in sera of NPC patients [78,79].

All human herpesviruses encode orthologues of deoxyuridine triphosphate nucleotide hydrolases (dUTPases), which are enzymes that catalyze the hydrolysis of dUTP to dUMP. While only alpha and gamma herpesviruses dUTPases share homology with the catalytic subunit of cellular dUTPases, all herpesviruses dUTPases share a novel domain of an unknown function [80]. EBV dUTPase (a product of the *BLLF3* gene) has been studied beyond its role in DNA replication with surprising results. BLLF3 binds to TLR2-MyD88 and activates the NFκB signaling pathway, increasing secretion of pro-inflammatory Th1 and Th17 cytokines and of IL-10 [81]. Anti-CD3 antibody induced-secretion of IFN-γ and proliferation of peripheral blood mononuclear cells (PBMCs) was inhibited by BLLF3, supporting negative regulation of T cells [82]. Although no studies have been conducted with whole virus and *BLLF3* knockout mutants, BLLF3 secretion in exosomes has been observed in stress-induced infected cells [83,84], and positive associations have been made in epidemiological studies between anti-BLLF3 antibodies and chronic fatigue syndrome [85,86]. Expression of the enzyme in clinical samples of Hodgkin lymphoma (HL), BL and NPC was found negative [87], while the group of Nichollas, J.M. et al. documented *BLLF3* expression in non-keratinizing undifferentiated NPC [88].

BPLF1 is a late structural protein that is part of the viral tegument [89,90]. BPLF1 exhibits multiple functions, during viral cell entry it intervenes with the uncoating of the capsid and helps the capsid passage through the nuclear pore complex; in the nucleus it acts as a deneddylase and as an ubiquitin-specific cysteine protease (USP) that interacts with host cullin 1 and cullin 4A promoting their degradation, and thus, inhibiting their E3 ubiquitin ligase activity [89,91]. This latter activity prevents host cell cycle S-phase progression creating a favorable environment for viral DNA replication. However, central to this review, is the capacity of BPLF1 to interfere with TLR signaling [92]. In the study by van Gent and collaborators, transfection of the *BPLF1* deubiquitinase catalytic subunit interfered with TLR activation of the NFκB signaling pathway, which correlated with interference of TLR3 induction of IFN-β and TLR2 induction of IL-8. BPLF1 achieved this function by removing TRAF6 and NEMO ubiquitin modifications [92]. Similar observations were previously made by Saito, et al. [93]. BPLF1 is among the EBV antigens whose humoral responses are good predictors of NPC risk and of disease stage stratification in a Taiwanese cohort [94]. Also, a *BPLF1*-knockout virus is approximately 90% less infective than the wild-type virus in humanized mice. The *BPLF1* mutant virus also exhibited a delayed and reduced cell transformation capacity and had a four-fold reduced capacity to develop DLBCL-like splenic tumors [95].

**Figure 1:**
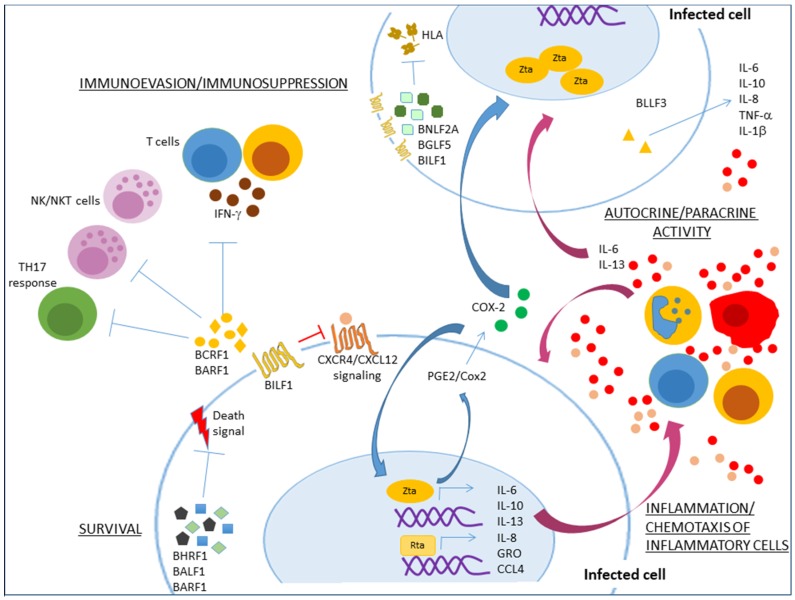
Lytic cycle proteins contribution to tumor initiation and tumor maintenance through enhanced survival, inflammation and immunomodulation. BHRF1 & BALF1, two viral orthologues of cellular Bcl-2, display anti-apoptotic functions protecting infected cells from death signals. Immunoevasion occurs through several mechanisms: BCRF1 (encoding a viral IL-10) and BARF1 contribute to reduced natural killer (NK) and cytotoxic T lymphocyte (CTL) responses as well as suppression of T cell activity through inhibition of IFN-γ. BILF1, BGLF5 and BNLF2a deregulate the HLA pathway to evade elimination of EBV infected cells. Also, BILF1 displays an interfering regulatory role upon CXCL12-dependent activation of CXCR4. Viral reactivation genes also importantly contribute to inflammation. Zta directly or indirectly enhances levels of IL-13, IL-8, CXC chemokine GRO, CCL4, IL-6, and IL-10. IL-6 and IL-10 are also increased by Rta. BLLF3 increases secretion of pro-inflammatory Th1 and Th17 cytokines and of IL-10. The resulting inflammatory microenvironment promotes tumor growth through autocrine/paracrine stimulation.

Zta is another EBV protein that inhibits antigen presentation through several different mechanisms: (i) through upregulation of *BILF1*, *BGLF5* and *BNLF2a* expression [96], (ii) more directly through transcriptional repression of CIITA, a cellular master regulator of HLA class II gene expression [97], and (iii) through transcriptional repression of CD74, the invariant chain that chaperons HLA class II molecules to cell surface [98]. Other Zta immune evasion mechanisms have been reported, for instance, Zta inhibited IFN-γ signaling and with this controlled CIITA expression preventing the IFN-γ-mediated HLA class II surface deposition [99]. Zta also prevented TNFα activation and TNFα-induced cell death [100]. BILF1 and SM, one of two open reading frames of the *BSLF2* early lytic gene, have also been shown to inhibit the RNA dependent protein kinase R (PKR) antiviral responses [62,101]. See Figure 1 for a cartoon of the activity of the anti-apoptotic and immunomodulatory lytic proteins.

## 5. An Autocrine/Paracrine Role of γ-Herpesvirus Inflammatory Mediators in Tumor Initiation and Maintenance

Experimental data obtained with lytic cycle deficient EBV mutants tested in humanized or immunodeficient murine models support a direct contribution of the viral lytic cycle in lymphomagenesis and independent from viral spreading [31,32]. Likewise, knockout/down of some of the EBV immunoevasins resulted in viruses with decreased capacity for in vitro B cell immortalization, in spite of an absent antagonistic host immune system [35,59,95]. It has been widely documented that inflammation favors cellular carcinogenesis and enhances tumor progression through promotion of cell proliferation, survival, stemness, invasion, metastasis, angiogenesis, among many other features of advanced neoplasias. EBV-induced neoplasias often occur in a background of inflammatory disease, for instance in HMB and HV, as well as in several primary immunodeficiencies [2]. Indeed, several of the EBV-induced neoplasias such as NPC, EBVaGC and HL are characterized by a dense infiltrate of immune cells in intimate interaction with tumor cells, that in HL constitutes up to 90% of the neoplasia. Also, lymphoepitheliomas of many tissues tend to be EBV positive, and those neoplasias are characterized by an extensive lymphocyte infiltrate.

Elevated levels of cytokines, chemokines and growth factors are commonly observed after viral reactivation. Zta alone directly enhanced levels of IL-8, growth-regulated oncogene (GRO) and macrophage inflammatory protein-1β (MIP-1β/CCL4) [102]. Transfection of either IE transcription factors *BZLF1* or *BRLF1* in 293 cells enhanced expression of cellular IL-6 and IL-10, and elevated levels of these cytokines are constitutively present in established LCLs [103]. Treatment with an inhibitory anti-IL-6 antibody inhibited growth of LCLs in SCID mice, and remission was observed in 8/12 patients with post-transplant lymphoproliferative disorder upon treatment with the anti-IL-6 antibody [104]. In a different study, Lee, C.H. et al. found that *BZLF1* transfection in an EBV negative NPC cell line resulted in an inflammatory secretome that included PGE2 [105]. PGE2 is a metabolite of COX-2, an important inflammatory enzyme. We have known for a long time that individuals with cardiovascular disease in anti-COX-2 prophylactic treatment exhibit lower incidence of several forms of cancer [106]. Elevated levels of COX-2 have been detected in EBV-induced NPC and LCLs, and Gandhi J. et al. observed that EBV reactivation in LCLs and BL cell lines occurred through activation of the COX-2/PGE2 pathway [107], supporting an autocrine positive regulatory loop in which Zta induces COX-2 expression and COX-2 further promotes a constitutive level of *BZLF1* transcription and viral reactivation. A similar paracrine/autocrine role has been proposed for IL-13. Like IL-6 and IL-10, IL-13 transcriptional promoter harbors Zta response elements. Katsamura K. R. et al. observed that wild type EBV was 10-fold more efficient than a *BZLF1* knockout mutant to transform PBMCs [108]. IL-13 was found responsible for such effect, since antibody-mediated blocking of IL-13 reduced the transformation efficiency of the wild type EBV, and recombinant IL-13 enhanced the efficiency of the mutant virus.

In addition to the anti-apoptotic and immunomodulatory capacity of some of the EBV lytic cycle proteins, lytic cycle expression has been associated with an inflammatory response that may be important for viral carcinogenesis or may help to sustain EBV transformed cells through autocrine mechanisms. For instance, a BARF1 autocrine mitogenic activity has been documented in transfected mouse fibroblasts, activity that was blocked with anti-BARF1 antibodies [109]. Likewise, sera of mice xenotransplanted with NPC cells or of NPC patients promoted in vitro cell growth, a mitogenic signal that was also inhibited with anti-BARF1 antibodies [110]. Ectopic expression of BARF1 enhances the tumorigenicity of NPC cell lines when transplanted into nude mice [111]. The 3-D structure of BARF1 exhibits similarities with CD200, a T cell co-stimulatory molecule. Whether BARF1 is a decoy of CD200 ligand, as it is of M-CSF, is unknown, but CD200 orthologues are encoded by other herpesviruses: Kaposi Sarcoma herpesvirus (KSHV) K14, human cytomegalovirus (HCMV) e127, human herpesvirus 6 (HHV6) and human herpesvirus 7 (HHV7) U85, and by adenovirus and poxvirus [112]. Likewise, _ebv_IL-10 shifts the cytokine profile of PBMCs protecting EBV infected cells from NK/NKT-mediated lysis [60]. The similitude between human and _ebv_IL-10 was noted since the initial cloning of the murine IL-10. The inhibitory activity of murine IL-10 over IFN-γ and Th1 responses called the attention of the research team, since it was known that IFN-γ inhibited EBV B cell immortalization [113]. As early as in this pioneer study, _ebv_IL-10 was considered a classic example of viral cytokine mimicry, although it was not until a few years later that the _ebv_IL-10 inhibition of IFN-γ was documented [114].

BILF1 is a surface G protein-coupled receptor (GPCR) that forms heterodimers with various human chemokine receptors expressed on B cells [115]. An interfering regulatory role for BILF1 upon CXCL12-dependent activation of CXCR4 has been observed, which may also be true for other chemokine receptors bound by BILF1 [116]. A purified form of BLLF3 in contact with resting PBMCs resulted in rapid release of TNFα, IL-1β, IL-10, IL-6 and IL-8 [82]. Depletion of monocytes from the PBMCs pool with an anti-CD14 antibody abolished the BLLF3 triggering of inflammatory cytokines, while a fraction enriched with only the CD14 population secreted similar levels of the inflammatory cytokines as the whole PBMCs. It is likely that BLLF3 activates this inflammatory secretome through the TLR2/Myd88/NFκB pathway, since anti-TLR2 antagonistic antibodies and a Myd88 dominant negative inhibited the secretion of IL-6 [81]. Although this could be seen as classical recognition by Toll receptors and part of the host’s antiviral responses, we have previously mentioned the autocrine/paracrine role of IL-6 to sustain LCLs growth and post-transplant lymphoproliferations [103,104]. 

KSHV is also a γ-herpesvirus whose viral life cycle also alternates between latent and lytic stages, with the latter further subdivided into immediate early, delayed early and late gene expression [117]. Rta/Open Reading Frame 50 (ORF50) is the transcription factor in charge of KSHV reactivation. Like EBV, KSHV has been associated with several human neoplasias: Kaposi sarcoma (KS), multicentric castleman disease (MCD) and primary effusion lymphoma (PEL). Inflammation promoting tumor initiation and progression seems particularly true for KSHV-induced neoplasias. For instance, KS originates in areas of traumatized skin [118], anti-retroviral therapy-induced immune reconstitution inflammatory syndrome (IRIS) favors development of KS and MCD [119,120], and Th1 cytokines promote KSHV persistence in B cells and monocytes [121]. Furthermore, KSHV encodes one of the largest cargos of immunomodulatory genes than any other human virus, most of which are expressed during the lytic cycle of infection but that are still central for survival of the infected cell and for viral tumorigenesis in mice. For instance, among the lytic cycle genes, KSHV encodes at least four interferon regulatory factors (vIRFs), a deubiquitinase that inhibits activation of RIG-1 (ORF64), an inducer of IRF7, TLR3 and Myd88 degradation (Rta/ORF50), and an inhibitor of cGAS-STING activation (ORF52); an inhibitor of the inflammasome, of IL-1β and IL-18 production and of cell death by pyroptosis (ORF63); a complement regulatory protein (KCP/ORF4); multiple genes that inhibit antigen presentation (e.g., K3, K5 & K14 (vCD200/OX2, equivalent to EBV BARF1)); several proteins that interfere with chemotaxis and/or activation of macrophages and other innate immune cells (e.g., vCCL1/vMIP-1, vCCL2/vMIP-2 and vCCL3/vMIP-3), which skew T cells towards Th2 responses; a _kshv_GPCR that functions as an orthologue of the human IL-8 receptor (ORF74); KI that encodes a constitutive active receptor with an Immunoreceptor Tyrosine-Based Activation Motif (ITAM); K15 that binds to TNF receptor-associated factors (TRAFs), constitutively activates MAPKs and NFκB and expression of cellular cytokines IL-1α, IL-1β, IL-6, IL-8, CCL2, CCL20, CXCL3 and COX-2; and finally, KSHV encodes an orthologue of cellular IL-6 (vIL-6). Furthermore, KSHV induces expression of multiple angiogenesis and lymphangiogenesis factors that are critical for the clinicopathological features that characterize KS and MCD lesions. KSHV immunomodulation is reviewed in [122].

KS is considered an inflammatory angioproliferative neoplasia, most likely originated from endothelial cells infected with KSHV. Like EBV, KSHV also activates a transient abortive lytic cycle immediately after infection characterized by expression of several immunomodulatory genes and without expression of genes encoding DNA replication enzymes and structural proteins [123]. Transfection of murine bone marrow-derived endothelial cell precursors with a KSHV-bacterial artificial chromosome (KSHV-BAC) resulted in expression of a mix of latent and lytic genes without production of viral particles and without inducing cell cytopathic effects, supportive of an abortive lytic cycle [124]. While consecutive passage of infected cells usually results in loss of the viral episome [125], xenotransplantation of transfected cells into SCID/NOD mice induced highly vascularized KS-like sarcomas. Transcriptomic analysis of explanted murine KS-like tumors revealed a 15-fold increased expression of lytic genes, whilst expressing equal levels of latent genes when compared with transfected cells maintained in culture. Still, tumors did not show evidence of increase KSHV genome replication nor of viral particles production, again supporting of an enhanced abortive lytic cycle cooperating with tumor implantation. The transcriptional analysis also uncovered a strong lytic cycle-associated inflammatory and angiogenic gene expression signature that is also present in primary human KS samples [124]. Altogether, these studies support that in vivo episome maintenance, contrary to their loss in cell culture, and tumor growth result from the viral capacity to strongly module angiogenic/inflammatory host responses and that these activities are likely due to abortive lytic gene expression. 

Several of the immunomodulatory lytic cycle-associated KSHV genes exhibit oncogenic activity. For instance, knockdown of the lytic cycle gene *_kshv_GPCR* resulted in delayed tumor formation, while no differences were observed between wild type and knockdown virus to induce cell proliferation in culture [124]. *_kshv_*GPCR alone leads to immortalization of human umbilical vein endothelial cells (HUVEC) and secretion of endothelial growth factors with the capacity of autocrine/paracrine signaling [126]. Indeed, *_kshv_GPCR* knockdown resulted in decreased expression of several KSHV lytic cycle genes indicating autocrine mechanisms of transcriptional activation. Of note, targeting of *_kshv_*GPCR in the few tumor cells expressing this protein resulted in tumor regression [127]. In the study by Montaner, S. et al. endothelial cells stably expressing _kshv_GPCR were generated and transplanted into mice mixed with cells expressing KSHV bona fide latent genes in a ratio similar to the ones found in KS lesions. Although those _kshv_GPCR-expressing cells were a minor component of the tumor, targeting of those few cells in established tumors resulted in whole tumor regression. Thus, this study supports a _kshv_GPCR paracrine signaling critical for tumor maintenance and that occurs in the absence of expression of additional lytic genes and viral replication. *_kshv_GPCR* expression has been linked to secretion of VEGF, CXCL8, GM-CSF, ANGPT2, etc. (reviewed in [128]). Other lytic cycle genes with in vitro immortalization capacity are K1, vIL-6, vIRF1 and vIRF2. Also, rapamycin is recommended to treat KS developing in post-transplanted patients. Rapamycin targets the neoplasia without affecting the host versus graft immune response, and the KSHV lytic cycle gene (ORF45) was mapped as one of the main viral rapamycin targets [129]. ORF45 has also been shown to counteract interferon-mediated antiviral host immune responses [130]. 

Like KS, MCD is also driven by the autocrine activity of inflammatory and angiogenic viral and cellular cytokines, such as vIL-6 and IL-10 [131]. MCD is defined as an angiofollicular hyperplasia that also harbors KSHV in latent and lytic cycle [132]. MCD usually co-presents with KS in HIV positive immunocompromised patients, so that areas of MCD and areas of KS are often observed within the same lymph node [133]. MCD arises together with a severe inflammatory syndrome that often is the direct cause of the patient’s death [134]. Indeed, IL-6 overexpression itself drives the development of a MCD-like hyperplasia in mice [135]. Taken together these MCD studies support a strong contribution of lytic genes for KSHV tumorigenic activity, although it is not clear whether it is a terminal lytic cycle supporting latent infection of neighboring cells, it is an abortive lytic cycle in which expression of the inflammatory/angiogenic genes is enticed in a subset of the latently infected cells, or both mechanisms can co-occur. Up to 25% of the KSHV latently infected cells (LANA positive) also express Rta/ORF50 in MCD lesions [132]. Also, expression of vIL-6 and other immunomodulatory genes is often observed in KSHV-induced neoplasias.

## 6. An HCMV Oncomodulatory Role

HCMV is a β-herpesvirus that exhibits a wide range tropism that includes endothelial cells, epithelial cells, fibroblasts, but which is more frequently found persistently infecting cells of the myeloid lineage. HCMV has also developed complex interactions with hosts that go beyond the escape from the antiviral response. Several of the gene products involved in immunomodulation are not expressed during latency, and interestingly, some seem dispensable for viral replication, suggesting additional functions other than viral dissemination [136]. For instance, viral glycoproteins US2, US3, US6 and US11 inhibit HLA class I- and class II-dependent antigen presentation to T cells [136,137]. Cells lacking of HLA molecules are still susceptible to elimination by NK cells. To counteract this effect, virally encoded HLA class I homolog UL18 can bind to the NK cell inhibitory receptor NKG2A/CD94 avoiding NK cell-mediated lysis [138,139]. Also, HCMV gpUL40 stimulates expression of HLA-E a non-classical HLA protein that minimizes NK cell lysis [140]. HCMV also encodes a _cmv_IL-10 (*UL111A* gene) that displays a potent immunosuppressive effect. For instance, _cmv_IL-10 blocked the correct maturation of dendritic cells and promoted maturation of pro-tumoral M2 macrophages [141,142]. Other lytic cycle immunomodulatory genes are: anti-apoptotic IE1 (homolog of cellular Bcl-X1), IE2 (homolog of Flip) and UL37/vMIA; pUL144 a TNFR homolog, pUL128 a CC-like chemokine that modulates monocyte activity; and interestingly, HCMV encodes four GPCR-like proteins, US27, US28, UL33 and UL78.

HCMV does not transform cells in culture, and therefore, it is not considered a tumor virus. However, HCMV usually infects cells through integrins and growth factor receptors, proteins that tend to be upregulated in most tumor cells. Hence, an increased tropism of HCMV for established tumors has been proposed, and several studies have reported a high frequency of HCMV infection in several types of cancers. Originally proposed for glioblastomas, an HCMV enhancing role of tumor malignancy has been documented, particularly for brain and colon cancers. In this role, HCMV infects tumor cells and through expression of the set of immunomodulatory genes contributes with enhanced tumor cell proliferation, survival, immunosuppression, angiogenesis, and invasion, activities that have been defined as oncomodulation [143,144]. Even though different lines of evidence support that HCMV immunomodulatory mechanisms cooperate with tumor progression, this is still controversial since several studies have failed to confirm enrichment of HCMV sequences or of HCMV gene expression in tumor samples [145,146,147]. For instance, glioblastoma cells infected with HCMV exhibited an increased cell proliferation, angiogenic activity and invasion, properties that were bestowed on increased secretion of IL-6 and paracrine/autocrine activation of the STAT3 pathway, and which were abolished when IL-6 or STAT3 were inhibited [148]. Of note, increased expression of IL-6, IL-6 receptor or activated (phosphorylated) STAT3 correlated with poor survival of glioblastoma patients, and so does loss of the IL-6 negative regulator SOCS3 [149,150,151]. Infection of a p53 mutant mouse with murine CMV also induced glioblastomas, while a STAT3 inhibitor reversed this effect [152].

In the study by Soroceanu, L. et al. the HCMV increased secretion of inflammatory/angiogenic proteins was reproduced by the sole expression of the _cmv_GPCR US28 [148]. Surface expression of US28 in glioblastoma cells was observed and tumor cells were invasive in response to CCL5/RANTES. RANTES is one of the US28 ligands and an inflammatory chemokine whose expression is also induced by US28, revealing another oncomodulatory autocrine loop. Moreover, RANTES expression also correlated with poor clinical outcomes [148]. In agreement with a tumor-promoting role for US28, US28-expressing cells promoted tumorigenesis in immunodeficient mice [153]. US27, UL33 and UL78, the other HCMV GPCRs, also promiscuously interact with chemokines and promiscuously hetero-oligomerize with host cell chemokine receptors, dramatically reducing surface levels of them. Host GPCR surface depletion may help to counteract immune elimination (reviewed in [128]). However, HCMV GPCRs also exhibit constitutive activity and in the murine model of US28 ectopic expression, the tumorigenic activity of this protein was closely associated with its ligand independent G-protein associated constitutive activity [153].

Ectopic expression of US28 in LGR5+ intestinal epithelial stem cells induced development of colon cancer in transgenic mice [154]. Overexpression of CCL2/MCP-1, another US28 ligand, further expanded the pool of intestinal epithelial stem cells and increased the US28-mediated tumorogenicity. HCMV infection of CD133+ glioma stem cells has also been documented [155]. Infected stem cells showed an increased survival and altered differentiation activity that was mapped to immediate early HCMV proteins and to the IL-6-STAT-3 signaling pathway [156,157]. Besides IL-6, HCMV infection of endothelial cells or fibroblasts induced a copious secretome rich in endothelial growth factors, inflammatory mediators and several cellular proteases [158]. Functional assays showed that the isolated secretome of infected cells promoted endothelial cell survival and neovessel formation. HCMV also exhibits tropism for CD133 positive glioblastoma cancer stem cells, inducing them to secrete _cmv_IL-10 that in turn induces microglia precursors to differentiate into M2-like macrophages [142]. Clinical studies have shown that medulloblastomas are also infected with HCMV, and infection of medulloblastoma cell lines induced activation of COX-2 and PGE2 [159]. Valganciclovir inhibited PGE2 synthesis, reduced cell replication and tumor growth in mice xenotransplanted with HCMV positive medulloblastoma cells [159]. HCMV infection of human colorectal cancer cells also led to increased expression of COX-2 [160]. US28 has also been implicated in activation of COX-2 through the NFκB pathway in NIH-3T3 cells, and pharmacological inhibition of COX-2 resulted in decreased size tumors and reduced angiogenic activity in nude mice xenotransplanted with US28-3T3 cells [161]. A US28-directed siRNA also resulted in downregulation of VEGF expression in glioblastoma cells [148]. 

In a cohort of 75 glioblastoma patients, the median rate of overall survival was 33 months in those patients with low-grade HCMV infection and 13 months in those with high-grade HCMV infection, while the median rates of 2-year survival were 63% and 17%, respectively [162]. An exploratory analysis of 22 glioblastoma patients treated with valgancyclovir for more than 6 months reported an increased 2-year survival rate of treated patients versus untreated similar stage controls (median overall survival of 24.1 versus 13.7 months) [163,164]. Overall survival at four years was 27.3% in anti-HCMV treated patients versus 5.9% in controls. Taken together all these studies argue that HCMV infection increases the malignancy features of already established tumors, and that it does it through a combination of mechanisms that include an induced inflammatory and angiogenic secretome that paracrinally activates neighboring cells promoting immunoscaping from T cell and NK cell cytolytic activity. Most of the viral genes responsible for this effect are expressed within the lytic cycle, nonetheless, their expression happens in the absence of cell lysis, and on the contrary, increased proliferation/survival of infected tumor cells is observed.

## 7. Conclusions

**Figure 2:**
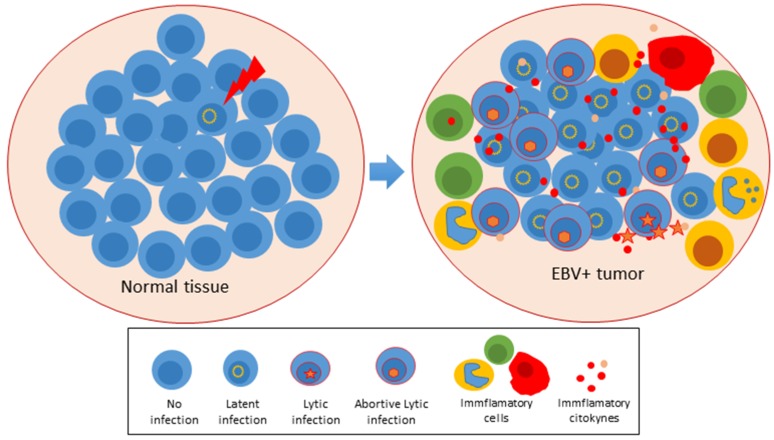
The cell transformation mediated by EBV is dependent on latent, lytic and abortive lytic infection. Evidence supports that lytic cycle gene expression contributes to the EBV-induced tumorigenesis. In EBV positive tumors, the abortive lytic cycle expression induces an inflammatory microenvironment that nurtures the tumor. This inflammatory microenvironment promotes tumor growth, survival and angiogenesis.

Mounting evidence supports that lytic cycle gene expression contributes to the EBV-induced tumorigenesis. However, it is still unclear whether it is complete or partial and whether the main point is to yield infectious viral particles or there is a direct input of oncogenic mechanisms. A complete lytic cycle seems incompatible with cell immortalization since it kills the infected cell, and indeed, the best-known EBV oncogenes are latent genes. However, EBV and related herpesviruses KSHV and HCMV encode a battery of immunomodulatory and anti-apoptotic lytic genes that are consistently expressed in LCLs and cancer samples. It is not unreasonable to think that viral persistency also depends on the expression of these genes and that tumors may also exploit their functions for sustained growth and survival. In this scenario, infected tumor cells may benefit with the capacity of lytic genes to downregulate HLA molecules, to counteract external and internal death stimuli, to manipulate autophagy, while the whole tumor may nurture with the viral capacity to organize a stroma enriched with immune cells and endothelial cells fulfilling pro-tumoral functions. This latter capacity was illustrated thorough the text with examples of infected cells secreting inflammatory and angiogenic molecules, such as IL-6, IL-10, IL-13, VEGF, and PGE2, but there is evidence in the literature of multiple other components of the virally-induced secretome, such as TGF-β, TNFα, IL-1β, etc. (Figure 2).

It is interesting to note that the EBV and KSHV immunomodulatory genes are mostly expressed early during the lytic cycle, while in HCMV several of them are late structural proteins. Indeed, for HCMV is more difficult to distinguish between a complete and a partial lytic cycle. Still, both mechanisms are not mutually exclusive and some cells in which reactivation mechanisms were initiated may return to a bona fide latent expression program, while others may reach the final stages of lytic expression. There are important clinical consequences from these mechanisms, for instance, HCMV seems more susceptible than both KSHV and EBV to the acyclovir family of antiviral drugs, even in already established tumors. On the contrary, it is clear that acyclovir therapy is not an option for EBV and KSHV cancer patients. Several EBV induced neoplasias are associated with increased humoral responses against terminal lytic genes, such as in NPC and EBVaGC, which could be explained by increased viral reactivation in B cells leading to increased infection of the epithelia. Moreover, levels of these anti-lytic protein antibodies positively correlate with levels of C-reactive protein and IL-6 supporting a close link between viral reactivation and inflammation [165]. This complete lytic cycle appears more prominent during the early stages conducting of carcinogenesis, and patients at risk of developing NPC or EBVaGC may benefit from antiviral therapy during pre-cancerous stages.

Also, worth mentioning is that several of the EBV early lytic genes interfere with cellular DNA damage responses, for instance BPLF1 [166,167], BGLF5 [168], BGLF4 [169] and Zta [170]. Thus, the presence of these products may increase the chance of viral tumorigenesis. Future studies should aim to identify authentic abortive-lytic genes, the mechanisms leading to their expression and their contribution to viral persistency and viral tumorigenesis. Naked unmethylated viral DNA explains transient post-infection expression of Zta, but how abortive expression happens in hypermethylated chromatinized DNA is not clear. Finally, there are several proteins and non-coding RNAs expressed as latent genes that also have immunomodulatory functions but which are beyond the scope of this review. However, the expression of lytic genes in established lymphomas and carcinomas should also invite us to re-evaluate the almost dogmatic view of latent gene expression understood as four different and almost exclusive programs leading to different mechanisms of viral carcinogenesis, since there is mounting evidence of tumors with variegated expression. For instance, Burkitt lymphomas with latency III programs, NPCs and EBVaGCs without LMP1 or LMP2A or without both. Also, BARF1 seems more consistently expressed in NPCs that the latter latency II proteins, and so is BNLF2a in EBVaGCs. Perhaps, only latency 0 and latency I represent the true viral mode of latent infection, while latency II and latency III together with expression of additional lytic genes should be widely recognized by the immune system and may be the battleground between virus and host. Nonetheless, it is important to continue to study the viral molecules and mechanisms that regulate viral persistency either latent, abortive or full lytic, aiming to develop better antiviral strategies that may serve to target EBV-, KSHV- and HCMV-induced pathogenesis.

## Figures and Tables

**Table 1:** Anti-apoptotic and immunomodulatory genes from Epstein-Barr virus (EBV) and their Kaposi Sarcoma herpesvirus (KSHV) and human cytomegalovirus (HCMV) orthologues.

EBV	KSHV	HCMV	Function
BHRF1	KSHV BCL-2	IE1	Anti-apoptotic
BALF1			Anti-apoptotic
BARF1	K14	e127	Anti-apoptotic, Paracrine/autocrine role in inflammation and immunomodulation, oncogene
BCRF1 (ebvIL-10)		cmvIL-10	Immunomodulatory, immunosuppressive
BILF1	ORF74 (kshvGPCR)	US27, US28, UL33 & UL78	Paracrine/autocrine role in inflammation and immunomodulation, immunoevasin
BGLF5			Immunomodulatory, immunosuppressive, immunoevasin
BNLF2A	K3 & K5	US2, US3, US6 & US11	Immunomodulatory, immunoevasin
BLLF3	ORF54	UL72	dUTPase, Paracrine/autocrine role in inflammation and immunomodulation
BPLF1	ORF64	UL48	Deneddylase, ubiquitin-specific cysteine protease, immunomodulatory
BZLF1 (Zta)			AP1 orthologue, paracrine/autocrine role in inflammation and immunomodulation
BILF1			Immunoevasin
BRLF1 (Rta)	Rta/ORF50		Paracrine/autocrine role in inflammation and immunomodulation

## Abbreviations

The following abbreviations are used in this manuscript:

Bcl-2	B cell lymphoma 2
BCR	B cell antigen receptor
BL	Burkitt lymphoma
CMV	cytomegalovirus
COX-2	cyclooxygenase-2
CTL	cytotoxic T cell
DLBCL	diffuse large B cell lymphomas
dUTPases	deoxyuridine triphosphate nucleotide hydrolases
E	early
EBER	EBV-encoded small RNA
EBNA	Epstein-Barr Nuclear Protein
EBV	Epstein-Barr Virus
EBVaGC	EBV associated gastric cancers
GPCR	G protein-coupled receptor
GRO	growth-regulated oncogene
HCMV	human CMV
HDAC	histone deacetylase
HHV6	human herpesvirus 6
HHV7	human herpesvirus 7
HLA	human leucocyte antigen
HMB	hypersensitivity to mosquito bites
HL	Hodgkin lymphoma
HUVEC	human umbilical vein endothelial cells
HV	hydroa vacciniforme
IE	immediate early
IL	interleukin
IRF	interferon regulatory factor
IRIS	immune reconstitution inflammatory syndrome
ITAM	Immunoreceptor Tyrosine-Based Activation Motif
KCP	KSHV complement regulatory protein
KS	Kaposi sarcoma
KSHV	Kaposi Sarcoma herpesvirus
L	late
LCLs	lymphoblastoid cell lines
LP	leader protein
LPD	lymphoproliferative disease
MAPK	mitogen activated protein kinase
MCD	multicentric castleman disease
miRNA	micro RNA
NK	Natural Killer
ORF	Open Reading Frame
PBMC	peripheral blood mononuclear cell
PEL	primary effusion lymphoma
PGE2	prostaglandin E2
PKC	protein kinase C
PKR	protein kinase R
PLC	phospholipase C gamma
sBARF1	soluble BARF1
shRNAs	small hairpin RNAs
TAP1	peptide transporter associated with antigen processing 1
TGFβ	transforming growth factor-β
TLR	Toll-like Receptor
TPA	12-*O*-tetradecanoylphorbol-13-acetate
TRAF	TNF receptor-associated factors
USP	ubiquitin-specific cysteine protease
VCA	viral capsid antigen
